# Patient-oriented gene set analysis for cancer mutation data

**DOI:** 10.1186/gb-2010-11-11-r112

**Published:** 2010-11-23

**Authors:** Simina M Boca, Kenneth W Kinzler, Victor E Velculescu, Bert Vogelstein, Giovanni Parmigiani

**Affiliations:** 1 Department of Biostatistics, Johns Hopkins Bloomberg School of Public Health, 615 N. Wolfe Street, Baltimore, MD 21205, USA; 2 Ludwig Center for Cancer Genetics and Therapeutics and Howard Hughes Medical Institute, Johns Hopkins Kimmel Cancer Center, 1650 Orleans Street, Baltimore, MD 21231, USA; 3 Department of Biostatistics, Harvard School of Public Health and Department of Biostatistics and Computational Biology, Dana-Farber Cancer Institute, 44 Binney Street, Boston, MA 02115, USA

## Abstract

Recent research has revealed complex heterogeneous genomic landscapes in human cancers. However, mutations tend to occur within a core group of pathways and biological processes that can be grouped into gene sets. To better understand the significance of these pathways, we have developed an approach that initially scores each gene set at the patient rather than the gene level. In mutation analysis, these patient-oriented methods are more transparent, interpretable, and statistically powerful than traditional gene-oriented methods.

## Background

To date, the sequences of all coding exons (the exome) have been determined in 74 cancers [1-8]. These studies have revealed that advanced cancers each generally harbor between 30 and 80 point mutations or small insertions or deletions. Other genetic alterations, such as amplifications and homozygous deletions, contribute another ten genes per tumor. These alterations can be categorized into two classes: 'drivers', which bestow a growth advantage on the cancer cell, inhibiting cell death or promoting cell birth and 'passengers' which coincidentally occurred in a cell that later or concurrently developed a driver mutation, but had no effect on cell proliferation. These same studies have defined a landscape consisting of both 'mountains' - drivers which are mutated at high frequency in tumors of the same type - and 'hills' - drivers which are mutated at low frequency in these tumors. Most driver genes appear to be hills, making it difficult or impossible to distinguish them from passenger mutations on the basis of frequency alone. A variety of bioinformatic studies based on these data have suggested that the mountains and hills, though heterogeneous among tumors, can be grouped into a much smaller set of pathways and biologic processes called 'gene sets.' This has led to the idea that an analysis of gene sets constituting these pathways and biologic processes may provide more information about the pathways altered in cancers than an analysis of individual genes.

Sequencing studies completed prior to those involving large scale sequencing have additionally revealed an 'exclusivity principle'. Within a single pathway, it is rare for multiple genes to be altered in a single tumor. Thus a tumor with a *KRAS *mutation generally does not also harbor a *BRAF *mutation, as *KRAS *is upstream of *BRAF *in the same pathway [9]. Similarly, *PIK3CA *and *PTEN *mutations do not generally occur in the same tumor, and other genes in the same pathway are also mutually exclusive [10]. The explanation for this principle is that once a mutation alters a pathway, the selective growth advantage incurred by a second mutation in the same pathway is minimal. Large scale sequencing studies of cancers [4,11,12] have provided additional support for the exclusivity principle.

Recent cancer genome projects have therefore evaluated gene sets in addition to scoring genes based on the number and types of alterations observed. Many methods have been proposed for this statistical task, beginning with [13] and [14] and reviewed in [15]. Most of these methods are gene-oriented, in that they first calculate a score for each gene while assigning each gene to a particular gene set. The next step is to determine which gene sets carry better scores than predicted by chance. In mutation analysis this also involves normalizing for the number of genes in each gene set and the sizes and nucleotide compositions of each gene in each gene set.

Gene set analysis was originally devised to evaluate expression data and when applied to mutational data, has not yet taken into account the exclusivity principle. By summarizing the data across patients one gene at the time, conventional gene set approaches are not able to differentiate between two very different scenarios. For example, consider a gene set including ten genes, and suppose ten different mutations are found among ten cancers of the same type. In one scenario, each of the ten genes might have a single mutation and, consistently with the exclusivity principle, one mutation may occur in each of the ten patients. In another scenario, one cancer might have mutations in all ten genes in the pathway, and the other nine cancers might each have no mutations. Conventional gene set analysis methods, which focus only on the number of alterations in a gene among the ten patients, cannot distinguish between these two scenarios. However, from a biological standpoint, in the former case, all patients have the relevant pathway altered while in the latter, only one patient has any mutations in this pathway.

We here describe a patient-oriented approach that factors in the principles noted above. Basically, we compute a patient-based score for each gene set. In its simplest version, this score can only have two values - one or zero (that is it is binary): the score is one if any gene within the gene set is altered in the individual patient's tumor and zero if no gene is altered. Though a strict interpretation of the exclusivity principle should preclude two genes in the same gene set from being altered in the same tumor, the present state of genome annotation is imperfect and also many genes are components of more than one gene set. Our approach allows us to cope with this imperfection while maintaining statistical rigor. We believe that this new analytic method more accurately reflects the selective pressures that drive mutation acquisition in naturally occurring cancers. Our assumption provides a unifying theme for organizing the mutations but there are exceptions, such as *PIK3CA *and *PTEN *in endometrial cancers. These exceptions are currently a weakness in our patient-centric model, but we believe they are uncommon. We note that other alterations, such as differences in copy number or epigenetic silencing, will be important to tumor growth, but our study is concerned only with the interpretation of the significance of genes that are altered through point mutations.

## Results and discussion

### Gene set analysis tools

We developed a number of patient-oriented techniques and compared them to each other as well as to a standard gene-oriented approach for analysis of the same data and gene sets.

For the gene-oriented approach, we started out with gene-specific scores. For each gene, the total number of mutations across all tumor samples was compared to that predicted from the passenger mutation rate, providing a score. The genes were then ranked according to these scores, with the most mutated genes ranked highest. For each gene set, we then determined whether the ranks of the scores for the genes within that gene set were higher than predicted by chance. The details of the scoring we used for this analysis are described in the Methods section.

For the main patient-oriented approach, we calculated a score (*T_s_*) for each gene set, defining this score in the simplest way possible ( [3,11] and [12]): the score is the number of patients in which the gene set is altered. We then considered randomly assigned mutations for the null hypothesis. For example, suppose that one of the tumors contained 60 mutations. The 60 mutations were randomly assigned to 60 different genes. A similar permutation was performed for each tumor. We then determined the scores for each gene set, that is, the number of patients in which the gene set was altered by one or more of the randomly assigned mutations. Finally, we assessed whether the scores of gene sets containing the randomly assigned genes was statistically different from the scores calculated from the actual experimental data. We also considered three other variations on this method (one where the null hypothesis considers mutations obtained from estimated passenger probabilities and two where tumor heterogeneity is included in the *T_s _*score, using either a permutation or a passenger null). More detailed descriptions of the statistical methods, as well as mathematical proofs, are described in Methods and expanded in the Additional file 1.

### Experimental results

We analyzed the mutation data on glioblastoma multiforme (GBM) patients in [4] using the patient-oriented and gene-oriented methods. The gene set annotations came from the MetaCore database [16]. We considered 3,071 sets, having between 3 and 2,096 genes. There was substantial overlap between the set annotations (mean: 33 genes; median: 9 genes; interquartile range: 5 to 29 genes).

In the GBM experimental dataset, 1,454 of the 3,071 gene sets were altered in at least one sample. Of the 1,454 gene sets with at least one altered gene, the great majority (1,131, representing 78%) had only one alteration per sample, in accordance with the exclusivity principle. The composition of these 1,131 gene sets was somewhat similar to those of the total 3,071 gene sets (mean: 17 genes; median: 8 genes; interquartile range: 4 to 20 genes). In contrast, there were 323 gene sets which had two or more alterations and these tended to be considerably larger than the average gene set (mean: 167 genes; median 116 genes; interquartile range: 61 to 197 genes). Note that the exclusivity principle is incompatible with passenger mutations occurring in the same gene set as driver mutations; passenger mutations are more likely to occur in bigger genes and in larger gene sets by chance alone. It is also possible that some of the gene sets fail to obey the exclusivity principle because they encompass parts of multiple pathways or processes, or because of faulty annotations. Information gained from gene set analysis, whether it be patient-oriented or gene-oriented, will improve as biologic knowledge of the relevant processes and pathways continually improves.

**Figure 1:**
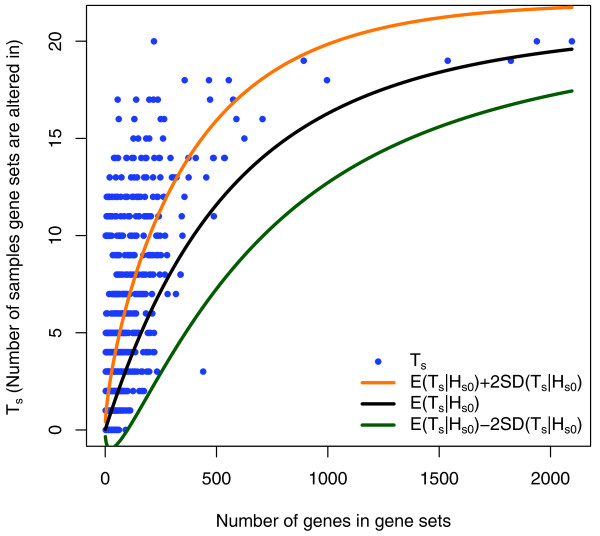
**Observed (blue) and expected number of altered samples (*T_s_*) across the gene sets in the dataset from **[4]**, as a function of the size of the gene set**. The expected numbers are computed using the permutation null and denoted by *E*(*T*_*s*_|*H*_*s*0_). The values within two standard deviations of the *E*(*T_s_*|*H*_*s*0_) are also shown.

**Figure 2:**
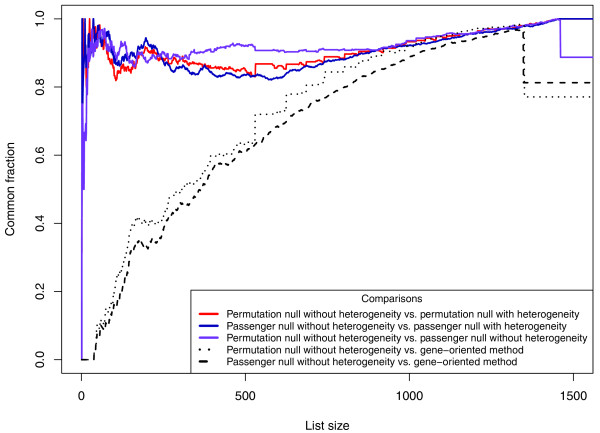
**CAT plot comparing the patient-oriented methods to the gene-oriented method for the glioblastoma data from **[4]. Each graph represents a pairwise comparison of two methods: The gene sets are ranked according to the *P*-value, a list of top gene sets is created at each rank, then the fraction of gene sets in the list common to both methods is graphed.

In Figure 1, a scatter plot of the number of GBM mutations per gene set (*T_s_*) vs the size of each gene set provides an overall sense of the variation. The expected number of mutations for each gene set under one of the null hypotheses (see Methods), as well as the values within two standard deviations of the expected number, are overlaid on these experimental data. These results show that mutations tend to cluster within gene sets far more than one would expect by chance. In particular, there were a large number of sets whose *T_s _*scores were more than two standard deviations away from the expected mean under the null hypothesis. Though this GBM study had only 21 subjects, it was still possible to get useful information at the gene set level for sets including as many as a few hundred genes; a larger number of tumors would have to be evaluated to get a statistically significant result for the largest gene sets. A more in-depth view of the dependence between the null distribution of *T_s _*and the size of the gene set is presented in Additional file 2. We developed and studied four implementations of the patient-oriented methods, differing only by the nature of the assumptions used to generate the null distributions and the normalization methods (see Methods). The patient-oriented methods and the gene-oriented method are further compared in Figure 2, which shows (using the CAT plot introduced in [17]) that the same gene sets are commonly identified by any two of the patient-oriented methods but that different gene sets are often identified by the gene-oriented method. (The plot shows only two of the patient-oriented methods being compared to the gene-oriented method, for the sake of clarity, but the remaining two display a similar behavior.)

Which approach is superior at identifying the 'true' gene sets involved in GBM? One way to address this question is through the evaluation of pathways known to be involved in this tumor type. Among the gene sets evaluated were those containing the *PI3K *or *RB1 *pathways, which are known to be altered in GBM. We would therefore expect that many of the gene sets containing the *PI3K *or *RB1 *pathways would be ranked high in any robust analysis of GBM mutational data. With the patient-oriented approach, we found all but one of the 15 gene sets containing *PI3K *or *RB1 *to be ranked at a high and statistically significant level (Additional file 3). In contrast, many gene sets containing the *PI3K *or *RB1 *pathways were not highly ranked with the gene-oriented method.

Another example of the superiority of the patient-oriented approach was provided by an analysis of the 106 gene sets containing *TP53*, the gene most frequently mutated in GBMs (10 of 21 samples). The presence of a mutation in a specific gene in a large fraction of tumors should implicate virtually any gene set containing that gene in the tumorigenic process. A gene set analysis with the patient-oriented method was in accord with this expectation, while an equivalent analysis with the gene-oriented method was not (all of the gene sets ranked among the top 50 contained *TP53 *when using the patient-oriented approach, with the exception of a single set in one of the four methods, whereas only four of the gene sets ranked among the top 50 contained *TP53 *when using the gene-oriented method). In the tumor subset in which *TP53 *was mutant, each of the top 52 sets (having ranks of at most 50, due to ties) included *TP53*, when analyzed with the main patient-oriented method. With the gene-oriented method, only ten of the top 50 gene sets included *TP53 *in those tumors in which *TP53 *was itself mutant.

We also looked at the sets which contained other candidate cancer genes (CAN-genes), as defined in [4], except for *CDKN2A *and *CDK4*, which were not mutated in any of the samples we considered. There were 411 sets in which at least one of these genes was present, but *TP53 *was not. Their median ranks were between 334 and 348 in each of the four patient-oriented methods.

Our methods permits sample size estimations for future studies which would draw from the same or a similar sample of tumors via simulation. We note that the number of patients required for significant results will vary from set to set, depending on the size of the gene set and the frequency with which it is altered. Consider for example the response to retinoic acid, which has the lowest *P*-value under the main patient-oriented method we consider, and is composed of only seven genes, of which *TP53 *and *LRP2*, both CAN-genes, are altered in a total of 12 samples. Out of 100 simulations performed in which 5 of the 21 patients were considered each time, 65 assign it a q-value of 0.1 or lower, which would often be considered significant. However, if we consider the set of genes involved in *PLAU *signaling in cell adhesion, which consists of 42 genes (three of which are CAN-genes) altered in seven samples, the simulation results would look very different. In the actual dataset, this gene set has a rank of 154.5, but nonetheless, is significant (q-value of 0.007). Out of 100 simulations with 5 samples, it only had a q-value less than or equal to 0.1 seven times; for 10 and 15 samples, this number went up to 42, respectively 76.

We also analyzed (see Additional file 4) data from three additional studies that comprehensively examined somatic mutations in breast, colorectal, and pancreatic tumors, reaching qualitatively similar conclusions.

### Controlled simulation results

To systematically examine the value of our method, we performed 100 simulations with the existing (3,071) gene sets presenting data consistent with a null distribution and other gene sets 'spiked-in,' that is having a high probability of being altered in varying proportions of the samples considered. These sets each have sizes of 25, 100, and 250 genes, with probabilities of being altered in a given patient of 0.25, 0.50, 0.75 and 0.90, resulting in 12 artificially generated sets. They were chosen to cover a wide range of gene set sizes and probabilities: For example, there were 260 sets containing between 20 and 30 genes, 124 sets containing between 75 and 125 genes, and 23 sets containing between 225 and 275 genes.

We considered two ways of generating null distributions in our simulations: one where the data on the genes present in the 'null' gene sets were obtained by permutation, and one where they were generated according to pre-specified mutation rates; further details are given in the Methods section. Using these simulated datasets, the patient-oriented approaches and the gene-oriented methods were compared with respect to sensitivity and specificity. In the analyses below, the spiked-in sets represent the biological signal, and if, discovered, are 'true positives.' The remaining gene sets represent the background noise, and if discovered, are 'false positives.'

#### Sensitivity (power)

**Figure 3:**
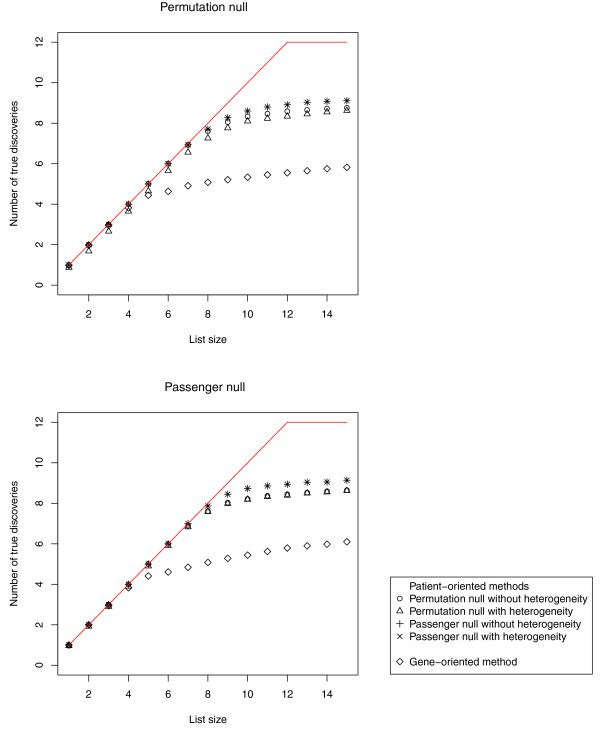
**Power analysis**. Plot of the average number of truly positive (spiked-in) gene sets included in the top X gene sets, as X varies. The red line indicates the ideal scenario over 100 simulation runs. Simulations use the permutation null (top panel) or the passenger null (bottom panel) data-generating mechanisms. The four patient-oriented methods return more true positives than the gene-oriented method, except when one focuses on short lists, which include sets that are relatively easy to detect. (Note that the overlap of the two methods which use the passenger null looks like a star (*).)

We compared the patient-oriented methods to the gene-oriented method in terms of the ranks of the spiked-in sets. The simulation results for the permutation null are presented in the top panel of Figure 3, showing how many spiked-in gene sets are identified within the top scoring sets. For example, for the primary patient-oriented approach, eight of the 12 spiked-in gene sets were among the top ten-scoring gene sets. Ideally, the plot would show a straight line for ranks between one and twelve, as indicated by the red segment. On average, the four patient-oriented methods identified more true positives for a given list size than the gene-oriented method.

Among the spiked-in gene sets considered, five are generally given good ranks by both the patient-oriented methods and the gene-oriented one. These gene sets tend to have both a relatively small number of genes and a high probability of being altered in any given patient, that is those with 25 genes and probabilities of being altered of 0.50, 0.75, and 0.90 and those with 100 genes and probabilities of being altered of 0.75 and 0.90. However, when the gene set size increases or the probability of being altered decreases, the patient-oriented methods perform better. For example, the gene set with 100 genes and a probability of being altered of 0.50 had median ranks of eight or nine across the 100 simulations for each of the patient-oriented methods, while for the gene-oriented method, the median rank was 28.5. Similar results were obtained with the gene set with 25 genes and probability 0.25 (the median rank of the patient-oriented approaches was between nine and ten, while for the gene-oriented approach it is 14), the gene sets with 250 genes and probabilities 0.90 (median ranks of five or six with the patient-oriented approaches versus 35 with the gene-oriented approach) and 0.75 (median ranks of seven or eight versus 201). The remaining three spiked-in gene sets generally did not achieve good ranks in either approach, due to the combination of the large number of genes and low probability of being altered; but again, the patient-oriented methods generally ranked them higher than the gene-oriented method.

An analogous graph for a second set of simulations which uses the pre-specified mutation rates to obtain the 'null' gene sets is presented in Figure 3, bottom panel. Results were qualitatively similar. For example, the gene set of size 250 and probability 0.75 got good median ranks with the patient-oriented methods (seven across the board), but a poor median rank with the gene-oriented approach (91).

We conclude that in simulation scenarios reflecting the exclusivity principle, patient-oriented methods have better power, especially for larger gene set sizes and lower probabilities of being altered.

#### Specificity

**Figure 4:**
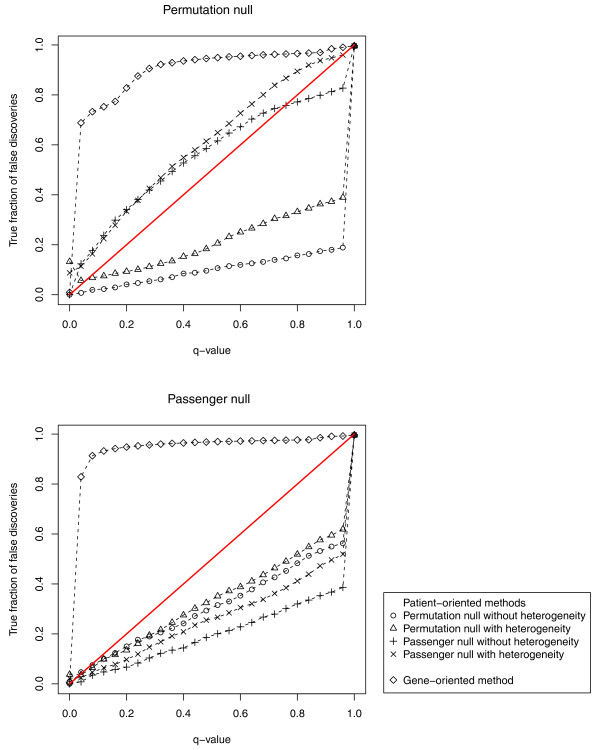
**Calibration analysis**. Plot of the average true fraction of false discoveries versus the average q-value over 100 simulation runs. The identity line indicates the ideal scenario of a perfect FDR-control rate; being above the identity line indicates anti-conservative behavior, and being below the identity line indicates conservative behavior. Simulations use the permutation null (top panel) or the passenger null (bottom panel). The gene-oriented method shows very anti-conservative behavior while the patient-oriented methods are generally calibrated or conservative.

We also considered how well the patient-oriented and gene-oriented approaches performed in terms of the number of false positives. Figure 4 shows plots of the actual true fraction of false positives versus the q-value for the 100 controlled simulations, employing the two different ways of generating null distributions (see above and Methods). In the ideal scenario the q-values would fall below the identity line, meaning that the q-values accurately control the false discovery rate (FDR). The patient-oriented approaches generally show appropriate behavior in both sets of simulations, as the true fraction of false discoveries is generally close to or much lower than the q-value. This is generally what is desired with an analytic method, as it lends confidence to the highest ranking gene sets. In contrast, the gene-oriented methods are anti-conservative: they identify a relatively large number of false positive gene sets, and are overly confident about the presence of false positives in the lists they generate. The two patient-oriented approaches that employ the passenger null are somewhat anti-conservative (though much less so than the gene-oriented approach) for the permutation null data-generating mechanism. This is due to the data-generating mechanism implying a higher rate of passenger mutations than is given by the actual estimated passenger rate, since the events being permuted include those in CAN-genes.

## Conclusions

We have developed and evaluated a patient-oriented approach for the gene set analysis of mutation data in cancer genome studies.

We performed simulations with spiked-in gene sets and applied the patient-oriented methods as well as the gene-oriented methods to their analysis. We found that the patient-oriented methods tended to outperform the gene-oriented methods in terms of power, especially when considering gene sets of a large size or low probabilities for alteration. We also appear to be gaining power when considering the real mutation data in [4], as many gene sets which had prior evidence of being altered received better scores/ranks with our approach than with the gene-oriented approach. Our new patient-oriented methods also performed better in terms of specificity (Figure 4).

Our results validate the analyses presented in [3] and [4] in three important ways: They provide a formal statistical evaluation of the properties and performance of patient-oriented gene set methods; they quantify the value added of patient-centered analyses over commonly used gene-centered analyses; and they confirm that in tumor studies including as few as 21 patients, there is significant power to detect relatively subtle signals as long as the analysis is carried out at the level of gene sets. This conclusion still applies to gene sets of relatively large size. The availability of complete exomic data from larger numbers of patients will make this approach even more valuable and accurate.

While we focused on mutation analysis, extensions of our methodology to consideration of other types of alterations measured on a genome-wide scale are possible. For example, it is straightforward to simply replace 'mutation' with 'any alteration' and proceed with the analysis approach proposed here. However, some of the null distributions required for statistical analysis would be more complex to generate with other types of alterations, such as copy number alterations (see Methods for a more detailed discussion). An important finding of our study was that the different patient-oriented methods we used generally yielded similar results, both for the controlled simulations and for the data in [4]. However, we recommend that the main method we considered (called the 'permutation null method without heterogeneity') is optimal, as it requires the least amount of data; in particular, it does not make use of gene coverages or of passenger rates. This is particularly important because passenger rates are extremely difficult to determine and may well vary from tumor type to tumor type and from patient to patient with the same tumor type. Compared to other gene set analysis techniques, we believe that the patient-oriented approaches are more transparent, more interpretable, and ultimately, ask questions of greater scientific interest.

## Materials and methods

### Gene set analysis

Our patient-oriented approach relies on starting with a statistic for each gene in each patient, which is either one or zero, depending on whether or not the gene is altered in the patient. (A possible extension may involve a gene-level statistic which represents a probability, and is thus between zero and one.) For each patient, we combine the statistics corresponding to all the genes in a specific gene set to get a binary gene set statistic, which is one if the gene set is altered in the patient and zero if it is not. We then obtain a statistic for each gene set by taking a weighted sum of the patient-based gene set statistics. We consider two possible types of weights, depending on whether or not the decision is made to incorporate tumor heterogeneity. In the case which does not account for heterogeneity, the gene set statistic is simply the number of patients in which the gene set is altered. We also consider two possible null hypotheses: either the events are permuted randomly within each patient, or they come from a 'passenger mutation rate' that is computed for each patient individually. Thus, we consider four different patient-oriented methods: permutation null without heterogeneity, permutation null with heterogeneity, passenger null without heterogeneity, and passenger null with heterogeneity. We are able to derive novel and explicit mathematical expressions for the *P*-values associated with the gene set test we propose. Formal definitions and proofs are given in the Additional file 1.

In gene expression applications, [18] and [15] advocated phenotype permutations as opposed to gene permutations, but a large part of their argument was based on the fact that microarray gene expression data is correlated. As we make use of mutation data, we do not expect this to be as important a consideration. We also note that we have only matched tumor-normal samples, so we cannot permute on the phenotypes.

For comparison, we use a gene-oriented method implemented in the *limma *package [19] and previously used in [3] (accessed through an interface developed for [20]). This implementation of the method first ranks genes according to a likelihood ratio test (LRT) using the null hypothesis that the genes are mutated according to the passenger rates, as detailed in [21] and [2]. For each gene set, the ranking of the genes within it is compared to the ranking of the genes outside it using the Wilcoxon test.

This method is similar to the GSEA method of [22], though it is much faster. It is possible to use other gene-specific scores in the Wilcoxon test besides the LRT score, such as the CaMP score, developed in [1]. There are also other statistical tests which can be applied to obtain a *P*-value for the gene sets. For example, a *t*-test may be used to compare the scores of the genes within a gene set to those of the genes outside it. We tried each of these variations on the glioblastoma dataset described in the next section, and the CAT plots comparing the patient-oriented to the gene-oriented methods were similar. Another approach could be to set a threshold, either on the number of mutations in a gene, or on another gene-specific score, then to create a contingency table for each gene set, comparing the number genes within and outside the set with the threshold. A test like the chi-squared test or Fisher's exact test could then be employed. This approach is often used in gene set analysis for expression data [15]. However, given that less than 5% of the genes are mutated in our dataset, any threshold would invariably result in over 95% of the gene-specific scores being below it, thus invalidating this approach.

In both our simulated and real data analysis we compute *P*-values for each approach considered. Starting from these *P*-values, we use the Benjamini-Hochberg approach for obtaining q-values [23] for FDR control. We note that this is not the ideal scenario for the application of this approach, since the null hypotheses are correlated and the test statistics for the different gene sets are discrete and not identically distributed. However, it is likely that these factors lead to q-values that are conservative.

### Experimental data

We consider the somatic nonsynonymous point mutations from the glioblastoma dataset in [4] which were present in the Discovery Screen. In this screen, tumor samples from 22 patients were initially analyzed, one of them being excluded from subsequent analysis due to treatment with temozolomide, which led to a very different mutation profile from the other tumors. Thus, only 21 samples were considered for further analysis in [4], as well as in the present study, with sequence and mutation data from 20,661 genes, from the CCDS, RefSeq, and Ensembl databases. We do not consider the copy-number alterations or the expression profiles, detected via microarrays, respectively serial analysis of gene expression (SAGE) on these samples in [4]. We note that the candidate cancer genes (CAN-genes) given in [4] were established using additional data from the examination of 21 of the genes mutated in the Discovery Screen in 83 additional glioblastoma tumors, comprising the Prevalence Screen; this data was also not considered in the current study. In the dataset considered in the current study, a total of 748 mutational events were present in 685 genes in 21 tumor samples. The gene with the most events was *TP53*, with 12 mutations in ten samples. The total number of events per tumor varied between 12 and 63 (median is 35), indicating some degree of heterogeneity between patients.

We use functional gene groups and pathways contained within the well-annotated MetaCore database [16], which includes metabolic pathways and signaling pathways, as well as other cellular functions and processes.

### Controlled simulations

We considered two kinds of data-generating mechanisms for the null distributions in our simulations: One where the data on genes present in the 'null' gene sets were obtained by a permutation, and one where they were generated according to prespecified mutation rates representing likely scenarios for passenger mutations. In the case of the permutation null, we started with the events in [4], excluded those from the known 'mountains' (*TP53, PTEN, RB1, EGFR*), and permuted the remaining mutations among genes, while choosing the context of mutations by using weights corresponding to the expected number of mutations given by the passenger rate.

After the null dataset was obtained by one of these two methods, we spiked in 12 gene sets, of sizes 25, 100, and 250 genes, with probabilities of being altered in a given patient of 0.25, 0.50, 0.75 and 0.90. We artificially created these gene sets using hypothetical genes. To generate realistic genes we sampled nucleotide compositions and sequencing coverage from the real genes in [4]. For each of the spiked-in sets and each sample, we generated a number from the Uniform(0,1) distribution to decide whether or not the gene set was altered in the respective sample (depending on whether the random number was larger or smaller than the probability of being altered). In both simulation scenarios, the mutated genes and their contexts were chosen so that on average, the proportions of mutations of different types are the same as they are among passengers.

## Abbreviations

CaMP score: cancer mutation prevalence score; CAN-gene: candidate cancer gene; CAT plot: 'correspondence at the top' plot; FDR: false discovery rate; GBM: glioblastoma multiforme; LRT: likelihood ratio test.

## Authors' contributions

SMB and GP designed the research; BV conceived of the idea; SMB performed the analysis and drafted the manuscript, with input from GP; SMB derived the theorems; SMB, KWK, VEV, BV, and GP interpreted the results and edited the manuscript. All authors read and approved the final manuscript.

## Supplementary Material

Additional file 1 **Notations, derivations, and theorems used for the patient-oriented methods**. Click here for file

Additional file 2 **Cumulative distribution functions (cdf) of *P*-values for the patient-oriented method with the permutation null without heterogeneity, for 10,000 null samples, using the number of events in the 21 glioblastoma samples from **[4]. Click here for file

Additional file 3 **Comparison of the main patient-oriented methods (passenger null without heterogeneity) to the gene-oriented method on the dataset in **[4]. Click here for file

Additional file 4 **Analyses on three more datasets**. Click here for file

## References

[B1] Sjoblom T Jones S Wood LD Parsons DW Lin J Barber TD Mandelker D Leary RJ Ptak J Silliman N Szabo S Buckhaults P Farrell C Meeh P Markowitz SD Willis J Dawson D Willson JK Gazdar AF Hartigan J Wu L Liu C Parmigiani G Park BH Bachman KE Papadopoulos N Vogelstein B Kinzler KW Velculescu VE The consensus coding sequences of human breast and colorectal cancers. Science 2006 314 268 274 10.1126/science.1133427 16959974

[B2] Wood LD Parsons DW Jones S Lin J Sjoblom T Leary RJ Shen D Boca SM Barber T Ptak J Silliman N Szabo S Dezso Z Ustyanksky V Nikolskaya T Nikolsky Y Karchin R Wilson PA Kaminker JS Zhang Z Croshaw R Willis J Dawson D Shipitsin M Willson JK Sukumar S Polyak K Park BH Pethiyagoda CL Pant PV The genomic landscapes of human breast and colorectal cancers. Science 2007 318 1108 1113 10.1126/science.1145720 17932254

[B3] Jones S Zhang X Parsons DW Lin JC Leary RJ Angenendt P Mankoo P Carter H Kamiyama H Jimeno A Hong SM Fu B Lin MT Calhoun ES Kamiyama M Walter K Nikolskaya T Nikolsky Y Hartigan J Smith DR Hidalgo M Leach SD Klein AP Jaffee EM Goggins M Maitra A Iacobuzio-Donahue C Eshleman JR Kern SE Hruban RH Core signaling pathways in human pancreatic cancers revealed by global genomic analyses. Science 2008 321 1801 1806 10.1126/science.1164368 18772397 PMC2848990

[B4] Parsons DW Jones S Zhang X Lin JC Leary RJ Angenendt P Mankoo P Carter H Siu IM Gallia GL Olivi A McLendon R Rasheed BA Keir S Nikolskaya T Nikolsky Y Busam DA Tekleab H Diaz LA Jr Hartigan J Smith DR Strausberg RL Marie SK Shinjo SM Yan H Riggins GJ Bigner DD Karchin R Papadopoulos N Parmigiani G An integrated genomic analysis of human glioblastoma multiforme. Science 2008 321 1807 1812 10.1126/science.1164382 18772396 PMC2820389

[B5] Ley TJ Mardis ER Ding L Fulton B McLellan MD Chen K Dooling D Dunford-Shore BH McGrath S Hickenbotham M Cook L Abbott R Larson DE Koboldt DC Pohl C Smith S Hawkins A Abbott S Locke D Hillier LW Miner T Fulton L Magrini V Wylie T Glasscock J Conyers J Sander N Shi X Osborne JR Minx P DNA sequencing of a cytogenetically normal acute myeloid leukaemia genome. Nature 2008 456 66 72 10.1038/nature07485 18987736 PMC2603574

[B6] Mardis ER Ding L Dooling DJ Larson DE McLellan MD Chen K Koboldt DC Fulton RS Delehaunty KD McGrath SD Fulton LA Locke DP Magrini VJ Abbott RM Vickery TL Reed JS Robinson JS Wylie T Smith SM Carmichael L Eldred JM Harris CC Walker J Peck JB Du F Dukes AF Sanderson GE Brummett AM Clark E McMichael JF Recurring mutations found by sequencing an acute myeloid leukemia genome. New England Journal of Medicine 2009 361 1058 1066 10.1056/NEJMoa0903840 19657110 PMC3201812

[B7] Pleasance ED Stephens PJ O'Meara S McBride DJ Meynert A Jones D Lin ML Beare D Lau KW Greenman C Varela I Nik-Zainal S Davies HR Ordonez GR Mudie LJ Latimer C Edkins S Stebbings L Chen L Jia M Leroy C Marshall J Menzies A Butler A Teague JW Mangion J Sun YA McLaughlin SF Peckham HE Tsung EF A small-cell lung cancer genome with complex signatures of tobacco exposure. Nature 2010 463 184 190 10.1038/nature08629 20016488 PMC2880489

[B8] Pleasance ED Cheetham RK Stephens PJ McBride DJ Humphray SJ Greenman CD Varela I Lin ML Ordonez GR Bignell GR Ye K Alipaz J Bauer MJ Beare D Butler A Carter RJ Chen L Cox AJ Edkins S Kokko-Gonzales PI Gormley NA Grocock RJ Haudenschild CD Hims MM James T Jia M Kingsbury Z Leroy C Marshall J Menzies A A comprehensive catalogue of somatic mutations from a human cancer genome. Nature 2009 463 191 196 10.1038/nature08658 20016485 PMC3145108

[B9] Rajagopalan H Bardelli A Lengauer C Kinzler K Vogelstein B Velculescu V Tumorigenesis: RAF/RAS oncogenes and mismatch-repair status. Nature 2002 418 934 10.1038/418934a 12198537

[B10] Parsons DW Wang TL Samuels Y Bardelli A Cummins JM DeLong L Silliman N Ptak J Szabo S Willson JK Markowitz S Kinzler KW Vogelstein B Lengauer C Velculescu VE Colorectal cancer: mutations in a signalling pathway. Nature 2005 436 792 10.1038/436792a 16094359

[B11] Lin J Gan C Zhang X Jones S Sjoblom T Wood L Parsons W Papadopoulos N Kinzler K Vogelstein B Parmigiani G Velculescu V A multidimensional analysis of genes mutated in breast and colorectal and colorectal cancers. Genome Research 2007 17 1304 1318 10.1101/gr.6431107 17693572 PMC1950899

[B12] The Cancer Genome Atlas Research Network Comprehensive genomic characterization defines human glioblastoma genes and core pathways. Nature 2008 455 1061 1068 10.1038/nature07385 18772890 PMC2671642

[B13] Tavazoie S Hughes JD Campbell MJ Cho RJ Church GM Systematic determination of genetic network architecture. Nature Genetics 1999 22 281 285 10.1038/10343 10391217

[B14] Mirnics K Middleton F Marquez A Lewis D Levitt P Molecular characterization of schizophrenia viewed by microarray analysis of gene expression in prefrontal cortex. Neuron 2000 28 53 67 10.1016/S0896-6273(00)00085-4 11086983

[B15] Goeman J Buhlmann P Analyzing gene expression data in terms of gene sets: methodological issues. Bioinformatics 2007 23 980 987 10.1093/bioinformatics/btm051 17303618

[B16] Ekins S Nikolsky Y Bugrim A Kirillov E Nikolskaya T Pathway mapping tools for analysis of high content data. Methods Mol Biol 2007 356 319 350 16988414 10.1385/1-59745-217-3:319

[B17] Irizarry R Warren D Spencer F Kim I Biswal S Frank B Gabrielson E Garcia J Geoghegan J Germino G Multiple-laboratory comparison of microarray platforms. Nature Methods 2005 2 345 350 10.1038/nmeth756 15846361

[B18] Allison D Cui X Page G Sabripour M Microarray data analysis: from disarray to consolidation and consensus. Nature Reviews Genetics 2006 7 55 65 10.1038/nrg1749 16369572

[B19] Michaud J Simpson KM Escher R Buchet-Poyau K Beissbarth T Carmichael C Ritchie ME Schutz F Cannon P Liu M Shen X Ito Y Raskind WH Horwitz MS Osato M Turner DR Speed TP Kavallaris M Smyth GK Scott HS Integrative analysis of RUNX1 downstream pathways and target genes. BMC Genomics 2008 9 363 10.1186/1471-2164-9-363 18671852 PMC2529319

[B20] Schaeffer E Marchionni L Huang Z Simons B Blackman A Yu W Parmigiani G Berman D Androgen-induced programs for prostate epithelial growth and invasion arise in embryogenesis and are reactivated in cancer. Oncogene 2008 27 7180 7191 10.1038/onc.2008.327 18794802 PMC2676849

[B21] Parmigiani G Lin J Boca S Sjoblom T Kinzler K Velculescu V Vogelstein B Statistical methods for the analysis of cancer genome sequencing data. Johns Hopkins University, Department of Biostatistics Working Papers 2007 Paper 126 http://www.bepress.com/jhubiostat/paper126/

[B22] Subramanian A Tamayo P Mootha VK Mukherjee S Ebert BL Gillette MA Paulovich A Pomeroy SL Golub TR Lander ES Mesirov JP Gene set enrichment analysis: a knowledge-based approach for interpreting genome-wide expression profiles. Proc Natl Acad Sci U S A 2005 102 15545 15550 10.1073/pnas.0506580102 16199517 PMC1239896

[B23] Benjamini Y Hochberg Y Controlling the false discovery rate: a practical and powerful approach to multiple testing. Journal of the Royal Statistical Society B 1995 57 289 300 http://www.jstor.org/pss/2346101

[B24] Thomas M Taub A Calculating binomial probabilities when the trial probabilities are unequal. Journal of Statistical Computation and Simulation 1982 14 125 131 10.1080/00949658208810534

[B25] Lange K Numerical Analysis for Statisticians 1999 New York: Springer Verlag

