# Supplementary material for: Patient-oriented gene set analysis for cancer mutation data
Source: Genome Biol. 2010 Nov 23;11(11):R112. doi: 10.1186/gb-2010-11-11-r112 (PMC3156951; doi:10.1186/gb-2010-11-11-r112)
Supplement: Additional file 1 — Notations, derivations, and theorems used for the patient-oriented methods. [file gb-2010-11-11-r112-S1.pdf]

Appendix

Notation

The notation used in summarized in Table 1.

Table 1: Notation.

Indexes	$i = 1, \dots, I$	index over samples
	$g = 1, \dots, G$	index over genes
	$s = 1, \dots, S$	index over gene sets
Sample level	N_i	number of events in sample i
	$\mathbf{N} = [\mathbf{N_i}]_{\mathbf{1 \le 1 \le I}}$	
Gene level	X_{gi}	a statistic for gene g in sample i (binary)
	$\mathbf{X} = [\mathbf{X_{gi}}]_{\mathbf{1 \le g \le G, 1 \le i \le I}}$	
	p_{gi}	probability of gene g being altered in sample i if gene g were a passenger gene (also called *passenger probability*)
	$\mathbf{p} = [\mathbf{p_{gi}}]_{\mathbf{1 \le g \le G, 1 \le i \le I}}$	
gene set level	m_{gs}	indicator of whether gene g is in gene set s
	$m_{+s} = \sum_{g=1}^{G} m_{gs}$	number of genes in gene set s
	$\mathbf{M} = [\mathbf{m_{gs}}]_{\mathbf{1 \le g \le G, 1 \le s \le S}}$	
	$Z_{si} = 1 - \prod_{m_{gs}=1}(1 - X_{gi})$	score for gene set s in sample i
	$T_s = \sum_{i=1}^{I} a_{si} Z_{si}$	score for gene set s (where a_{si} are known positive constants)

We consider $X_{gi}, N_i, \mathbf{N}, \mathbf{T_s}$ and Z_{si} to be random variables, and denote their observed values by $x_{gi}, n_i, \mathbf{n}, \mathbf{t_s}$ and z_{si}, respectively. We assume that all the I samples are independent of each other. We also assume that all the G genes are independent of each other.

Preliminaries

Result We note that we can calculate both $E(T_s)$ and $Cov(T_{s1}, T_{s2})$ in terms of the means of X_{gi}.

Proof. We primarily use the assumption of independence of genes and samples.

$$
\begin{aligned}
E(Z_{si}) &= E\{1 - \prod_{m_{gs}=1} (1 - X_{gi})\} = 1 - \prod_{m_{gs}=1} \{1 - E(X_{gi})\} \\
=> E(T_s) &= \sum_{i=1}^{I} a_{si} E(Z_{si}) = \sum_{i=1}^{I} a_{si} - \sum_{i=1}^{I} a_{si} \prod_{m_{gs}=1} \{1 - E(X_{gi})\}
\end{aligned}
$$

Denote the intersection of the gene sets s_1 and s_2 by r. Denote by c_1 the difference between s_1 and r and

by c_2 the difference between s_2 and r.

$$\begin{aligned}
Z_{s_1,i}Z_{s_2,i} &= \{1-\prod_{m_{gs_1}=1}(1-X_{gi})\}\{1-\prod_{m_{gs_2}=1}(1-X_{gi})\} \\
&= \{1-\prod_{m_{gr}=1}(1-X_{gi})\prod_{m_{gc_1}=1}(1-X_{gi})\}\{1-\prod_{m_{gr}=1}(1-X_{gi})\prod_{m_{gc_2}=1}(1-X_{gi})\} \\
&= 1-\prod_{m_{gs_1}=1}(1-X_{gi})-\prod_{m_{gs_2}=1}(1-X_{gi}) \\
&+ \prod_{m_{gr}=1}(1-X_{gi})^2\prod_{m_{gc_1}=1}(1-X_{gi})\prod_{m_{gc_2}=1}(1-X_{gi}) \\
&= 1-\prod_{m_{gs_1}=1}(1-X_{gi})-\prod_{m_{gs_2}=1}(1-X_{gi}) \\
&+ \prod_{m_{gr}=1}(1-X_{gi})\prod_{m_{gc_1}=1}(1-X_{gi})\prod_{m_{gc_2}=1}(1-X_{gi}) \\
=> E(Z_{s_1,i}Z_{s_2,i}) &= 1-\prod_{m_{gs_1}=1}\{1-E(X_{gi})\}-\prod_{m_{gs_2}=1}\{1-E(X_{gi})\} \\
&+ \prod_{m_{gr}=1}\{1-E(X_{gi})\}\prod_{m_{gc_1}=1}\{1-E(X_{gi})\}\prod_{m_{gc_2}=1}\{1-E(X_{gi})\} \\
E(Z_{s_1,i})E(Z_{s_2,i}) &= \{1-\prod_{m_{gs_1}=1}\{1-E(X_{gi})\}\}\{1-\prod_{m_{gs_2}=1}\{1-E(X_{gi})\}\} \\
&= 1-\prod_{m_{gs_1}=1}\{1-E(X_{gi})\}-\prod_{m_{gs_2}=1}\{1-E(X_{gi})\} \\
&+ \prod_{m_{gr}=1}\{1-E(X_{gi})\}^2\prod_{m_{gc_1}=1}\{1-E(X_{gi})\}\prod_{m_{gc_2}=1}\{1-E(X_{gi})\} \\
=> Cov(Z_{s_1,i}, Z_{s_2,i}) &= E\{Z_{s_1,i}Z_{s_1,i}\}-E\{Z_{s_1,i}\}E\{Z_{s_1,i}\} \\
&= \{1-\prod_{m_{gr}=1}\{1-E(X_{gi})\}\} \\
&\quad \prod_{m_{gr}=1}\{1-E(X_{gi})\}\prod_{m_{gc_1}=1}\{1-E(X_{gi})\}\prod_{m_{gc_2}=1}\{1-E(X_{gi})\} \\
=> Cov(T_{s_1}, T_{s_2}) &= \sum_{i=1}^{I} a_{s_1,i}a_{s_2,i}Cov(Z_{s_1,i}, Z_{s_2,i})
\end{aligned}$$

□

In particular, we note that $Cov(Z_{s_1,i}, Z_{s_2,i}) \geq 0$, so $Cov(T_{s_1}, T_{s_2}) \geq 0$.

Binary Gene Statistic

The scenario we consider is where:

$$X_{gi} = \begin{cases} 1 & \text{if gene } g \text{ is altered in sample } i \\ 0 & \text{if gene } g \text{ is not altered in sample } i \end{cases}$$

Given that we are considering somatic mutations, we note that it is extremely rare to have more than one event per gene, so this definition is nearly equivalent to:

$$X_{gi} = \begin{cases} 1 & \text{if gene } g \text{ has one event in sample } i \\ 0 & \text{else} \end{cases}$$

We note that in this case, Z_{si} is an indicator of whether gene set s is altered in sample i. Thus, by definition, both X_{gi} and Z_{si} are Bernoulli random variables.

We denote the null hypothesis for gene set s by H_{s0}, and the distributions of Z_{si} and T_s under the null by Z^0_{si} and T^0_s. We note that there are a number of null hypotheses to choose from. We consider the "permutation" null and the "passenger rate" null, defined below.

Statement of Permutation Null

H_{s0} : The probability distribution of the number of samples gene set s is mutated in (T_s) is the same as it would be if we randomly chose I samples which had the same number of events as the samples under consideration ($N_i = n_i$ for $i = 1, \ldots, I$, or, equivalently, $\mathbf{N} = \mathbf{n}$), i.e., if we permuted the number of events within samples.

We take the "repeated draws of the I random samples" to be permutations of the events in the existing samples among the G genes. Since we assume that each gene can have at most one event per tumor sample, to get T^0_s, we choose n_i genes out of G genes to have 1 event each, for each of the I tumor samples.

Statement of Passenger Rate Null

H_{s0} : The probability distribution of the number of samples gene set s is mutated in (T_s) is the same as it would be if we randomly chose I samples which had the same passenger mutation rates as the samples under consideration.

Results under Permutation Null

We assume that under the permutation null, every gene has at most one mutational event per sample.

Theorem $Z^0_{si} \sim \text{Bernoulli}(q_{si})$, where $q_{si} = 1 - \frac{\binom{G-n_i}{m_{+s}}}{\binom{G}{m_{+s}}}$.

Proof. We get:

$$
\begin{aligned}
q_{si} &= E(Z^0_{si}) = P(Z^0_{si} = 1) = 1 - P(Z^0_{si} = 0) = 1 - P(\text{gene set } s \text{ is not altered in sample i} | \text{sample } i \text{ has } n_i \text{ events}) \\
&= 1 - P(\text{select } m_{+s} \text{ genes with 0 events in sample } i \text{ out of } G \text{ genes} \mid \text{sample } i \text{ has } n_i \text{ events}) \\
&= 1 - \frac{\binom{n_i}{0}\binom{G-n_i}{m_{+s}-0}}{\binom{G}{m_{+s}}} = 1 - \frac{\binom{G-n_i}{m_{+s}}}{\binom{G}{m_{+s}}}
\end{aligned}
$$

□

Note We can also use approximations of the hypergeometric distribution, such as $1 - \left\{\frac{G-n_i}{G}\right\}^{m_{+s}}$ or $1 - \left\{\frac{G-m_{+s}}{G}\right\}^{n_i}$, given that both m_{+s} and n_i are generally much smaller than G. The results will be nearly identical in all of the three cases.

Corollary Under the assumptions that under the permutation null, every gene has at most one mutational event and that all the events are independent of each other, T_s^0 is a weighted sum of independent Bernoulli random variables. We give exact results for $E(T_s^0)$, $Cov(T_{s1}^0, T_{s2}^0)$, and $Var(T_s^0)$.

Proof. Using the theorem above, Z_{si}^0 are independent Bernoulli random variables.

$$
\begin{aligned}
E(T_s^0) &= \sum_{i=1}^{I} a_{si} Z_{si}^0 = \sum_{i=1}^{I} a_{si} - \sum_{i=1}^{I} a_{si} \frac{\binom{G-n_i}{m_{+s}}}{\binom{G}{m_{+s}}} \\
Cov(Z_{s_1,i}^0, Z_{s_2,i}^0) &= \{1 - \prod_{m_{gr}=1} \{1 - E(X_{gi}|N_i = n_i)\}\} \\
& \quad \prod_{m_{gr}=1} \{1 - E(X_{gi}|N_i = n_i)\} \\
& \quad \prod_{m_{gc_1}=1} \{1 - E(X_{gi}|N_i = n_i)\} \prod_{m_{gc_2}=1} \{1 - E(X_{gi}|N_i = n_i)\} \\
&= \left\{1 - \frac{\binom{G-n_i}{m_{+r}}}{\binom{G}{m_{+r}}}\right\} \left\{ \frac{\binom{G-n_i}{m_{+s_1}+m_{+s_2}-m_{+r}}}{\binom{G}{m_{+s_1}+m_{+s_2}-m_{+r}}} \right\} \\
&= \frac{\binom{G-n_i}{m_{+s_1}+m_{+s_2}-m_{+r}}}{\binom{G}{m_{+s_1}+m_{+s_2}-m_{+r}}} - \frac{\binom{G-n_i}{m_{+r}}}{\binom{G}{m_{+r}}} \frac{\binom{G-n_i}{m_{+s_1}+m_{+s_2}-m_{+r}}}{\binom{G}{m_{+s_1}+m_{+s_2}-m_{+r}}} \\
=> Cov(T_{s_1}^0, T_{s_2}^0) &= \sum_{i=1}^{I} a_{s_1,i} a_{s_2,i} Cov(Z_{s_1,i}^0, Z_{s_2,i}^0) \\
&= \sum_{i=1}^{I} a_{s_1,i} a_{s_2,i} \frac{\binom{G-n_i}{m_{+s_1}+m_{+s_2}-m_{+r}}}{\binom{G}{m_{+s_1}+m_{+s_2}-m_{+r}}} \\
&- \sum_{i=1}^{I} a_{s_1,i} a_{s_2,i} \frac{\binom{G-n_i}{m_{+r}}}{\binom{G}{m_{+r}}} \frac{\binom{G-n_i}{m_{+s_1}+m_{+s_2}-m_{+r}}}{\binom{G}{m_{+s_1}+m_{+s_2}-m_{+r}}}
\end{aligned}
$$

In particular:

$$
Var(T_s^0) = \sum_{i=1}^{I} a_{si}^2 \frac{\binom{G-n_i}{m_{+s}}}{\binom{G}{m_{+s}}} - \sum_{i=1}^{I} a_{si}^2 \left[\frac{\binom{G-n_i}{m_{+s}}}{\binom{G}{m_{+s}}} \right]^2
$$

□

Results under Passenger Rate Null

Theorem $Z_{si}^0 \sim \text{Bernoulli}(q_{si})$, where:

$$
q_{si} = 1 - \prod_{m_{gs}=1} (1 - p_{gi}).
$$

Proof.

$$
\begin{aligned}
q_{si} &= E(Z_{si}^0) \\
&= 1 - \prod_{m_{gs}=1} \{1 - P(X_{gi} = 1|\mathbf{p})\} \\
&= 1 - \prod_{m_{gs}=1} (1 - p_{gi})
\end{aligned}
$$

□

Corollary We can similarly derive exact results for $E(T_s^0)$, $Cov(T_{s1}^0, T_{s2}^0)$, and $Var(T_s^0)$ in this case.

Calculation of P-values

For the case where $a_{si} = 1$ for all $1 \leq i \leq I; 1 \leq s \leq S$, we use the algorithm described in [24] to calculate the p-values. For the case where $a_{si} = 1$ does not always hold, we provide the theorem below.

Theorem We provide a recursive method for calculating the cdf of T_s^0.

Proof. We model this proof on the proof in Section 1.7 of [25].

We define the following random variables and consider their cdfs:

$$\begin{aligned} T_s^{0,j} &= \sum_{i=1}^{j} a_{si} Z_{si}^0 \text{ for } 1 \leq j \leq I, \\ F_s^{0,j}(x) &:= Pr(T_s^{0,j} \leq x). \end{aligned}$$

We can calculate $F_s^{0,j}$ recursively, beginning with $j = 1$:

$$\begin{aligned} F_s^{0,1}(x) &= 0 \text{ for } x < 0, \\ F_s^{0,1}(x) &= Pr(a_{s1} Z_{s1}^0 \leq x) = Pr(Z_{s1} = 0) = 1 - q_{s1} \text{ for } 0 \leq x < a_{s1}, \\ F_s^{0,1}(x) &= Pr(a_{s1} Z_{s1}^0 \leq x) = Pr(Z_{s1} = 0 \text{ or } Z_{s2} = 1) = 1 \text{ for } x \geq a_{s1}, \end{aligned}$$

since $a_{si} > 0$ for all $1 \leq i \leq I$.

For $j > 1$, $F_s^{0,j}$ can easily be calculated in terms of $F_s^{0,j-1}$:

$$\begin{aligned} F_s^{0,j}(x) &= Pr(\sum_{i=1}^{j} a_{si} Z_{si} \leq x) \\ &= Pr(\sum_{i=1}^{j} a_{si} Z_{si} \leq x | Z_{sj} = 1) P(Z_{sj} = 1) + Pr(\sum_{i=1}^{j} a_{si} Z_{si} \leq x | Z_{sj} = 0) P(Z_{sj} = 0) \\ &= Pr(\sum_{i=1}^{j} a_{si} Z_{si} \leq x - a_{sj}) P(Z_{sj} = 1) + Pr(\sum_{i=1}^{j} a_{si} Z_{si} \leq x) P(Z_{sj} = 0) \\ &= q_{sj} F_s^{0,j-1}(x - a_{sj}) + (1 - q_{sj}) F_s^{0,j-1}(x) \end{aligned}$$

In particular, for $j = I$, $F_s^{0,I}$ is the cdf of T_s^0. □

Corollary We can use the theorem above to calculate the p-value, which is equal to $P(T_s^0 \geq t_s)$, where t_s is the observed value of T_s, since $P(T_s^0 \geq t_s) = 1 - P(T_s^0 < t_s) = 1 - F_s^{0,I}(t_s-)$.

Tumor Heterogeneity

One may choose whether or not to incorporate tumor heterogeneity into the analysis. This can be reflected in the sample-specific constants a_{si}. If no tumor heterogeneity is assumed, then $a_{si} = 1$. If tumor heterogeneity is incorporated, a variety of options are available. We take $a_{si} = \frac{1}{q_{si}}$ in this case, so that, for each gene set, tumors which have a higher probability of being altered in the respective set under the null, get down-weighted. Thus, we consider 4 different methods: *permutation null without heterogeneity, permutation null with heterogeneity, passenger null without heterogeneity,* and *passenger null with heterogeneity.*
