# Supplementary material for: Patient-oriented gene set analysis for cancer mutation data
Source: Genome Biol. 2010 Nov 23;11(11):R112. doi: 10.1186/gb-2010-11-11-r112 (PMC3156951; doi:10.1186/gb-2010-11-11-r112)

Cumulative distribution functions (cdf) of p-values for the patient-oriented method with the permutation null without heterogeneity, for 10,000 null samples, using the number of events in the 21 glioblastoma samples from [4]. If T_s were continuous, rather than discrete, the p-value distribution under the null hypothesis would be Uniform(0, 1), and the cdf would be the identity line on the unit square. The degree of accuracy of the continuous approximation depends on the set size.

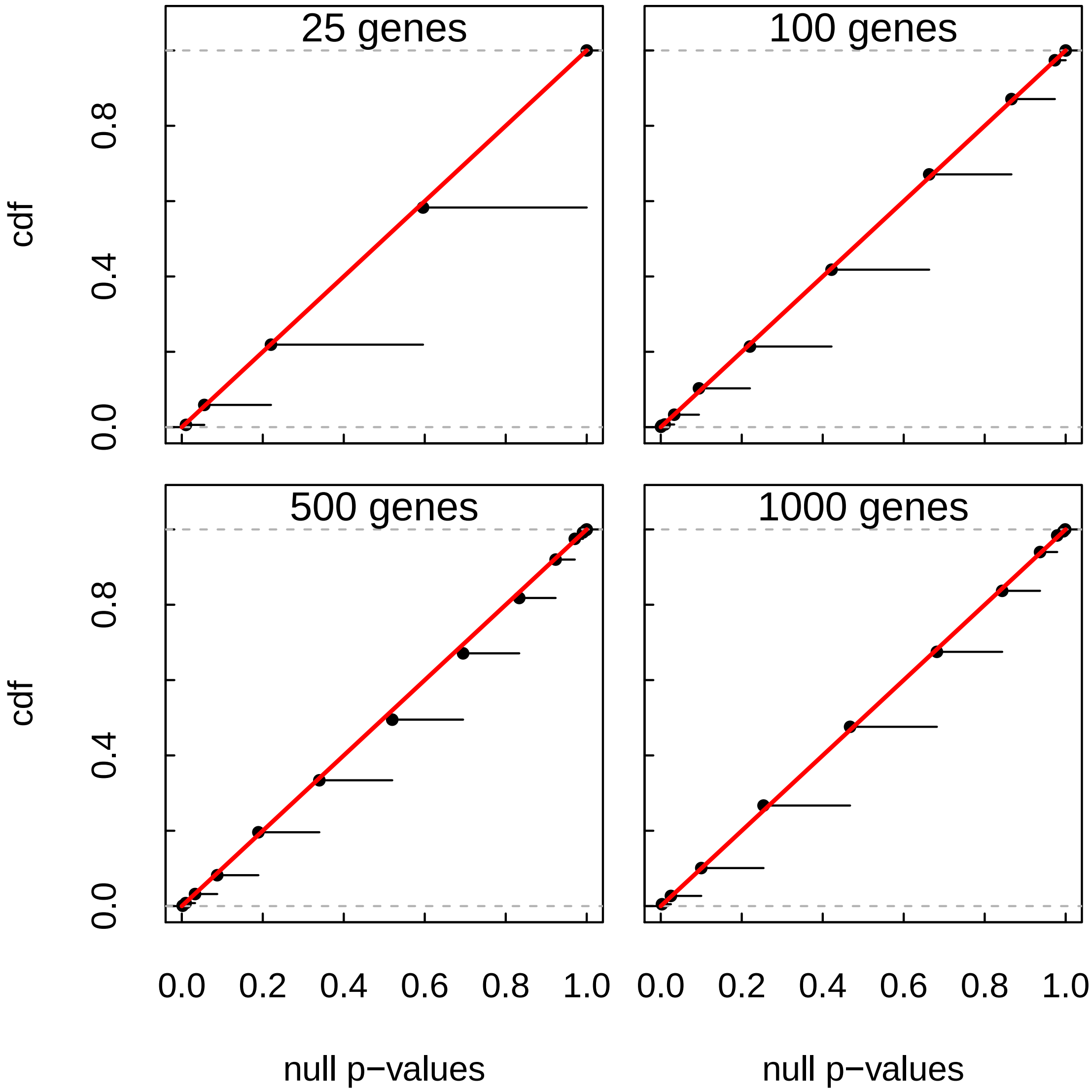

Supplement: Additional file 2 — Cumulative distribution functions (cdf) of P-values for the patient-oriented method with the permutation null without heterogeneity, for 10,000 null samples, using the number of events in the 21 glioblastoma samples from [4]. [file gb-2010-11-11-r112-S2.pdf]
