# Supplementary material for: Patient-oriented gene set analysis for cancer mutation data
Source: Genome Biol. 2010 Nov 23;11(11):R112. doi: 10.1186/gb-2010-11-11-r112 (PMC3156951; doi:10.1186/gb-2010-11-11-r112)

Comparison of the main patient-oriented methods (passenger null without heterogeneity) to the gene-oriented method on the dataset in [4]. The points in blue are gene sets which include genes in the *PI3K* pathway and the points in orange are gene sets which include genes in the *RB1* pathway. The vertical and horizontal lines are drawn at q-values of 0.1.

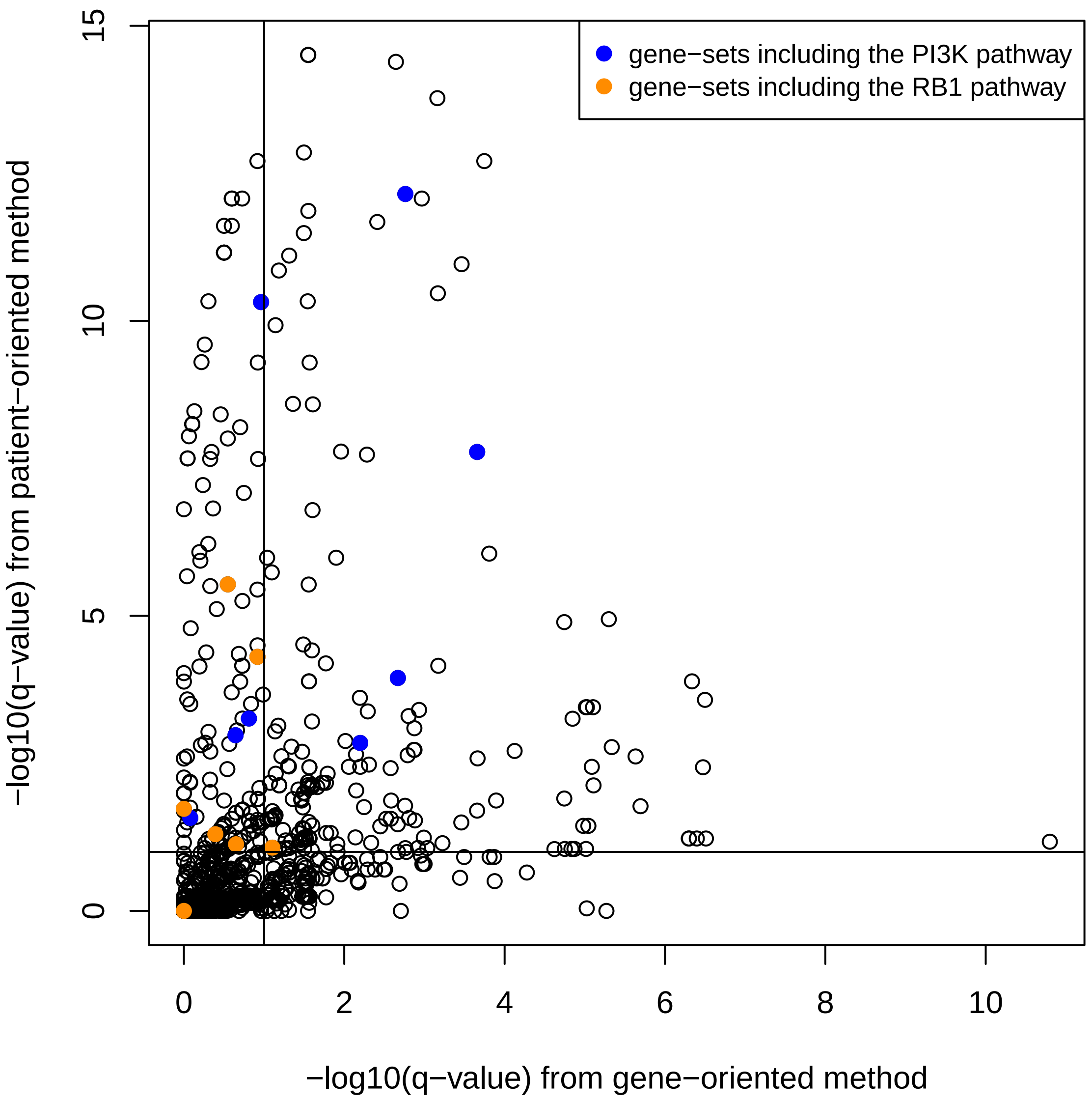

Supplement: Additional file 3 — Comparison of the main patient-oriented methods (passenger null without heterogeneity) to the gene-oriented method on the dataset in [4]. [file gb-2010-11-11-r112-S3.pdf]
