# Supplementary material for: Patient-oriented gene set analysis for cancer mutation data
Source: Genome Biol. 2010 Nov 23;11(11):R112. doi: 10.1186/gb-2010-11-11-r112 (PMC3156951; doi:10.1186/gb-2010-11-11-r112)

Additional Analyses

In addition to the analysis of the somatic point mutations from the glioblastoma Discovery Screen samples from [4], we also considered three additional datasets, for pancreatic cancer ([3]), and breast and colorectal cancers ([1], [2]). One again, we only considered the somatic point mutations identified in the Discovery Screen. For pancreatic cancer, 20671 transcripts from 20359 genes were analyzed in 24 samples, leading to 1163 mutations in 1007 genes. For breast and colorectal cancers, 18190 genes were analyzed in 11 samples, leading to 1112 mutations in 1026 genes, respectively 849 mutations in 769 genes. The MetaCore pathway database ([16]) was used for the set annotations. For the pancreas dataset, out of 3071 gene-sets, 1683 had at least one alteration, out of which 1280 had at most one alteration per sample, in accordance with the exclusivity principle. There were 366 genes which are present in the set annotations but were not present in the breast and colorectal cancer datasets. Thus, for those analyses, the set annotations used were modified to exclude those genes. For the breast cancer dataset, 1746 gene-sets had at least one alteration, 1189 of which had at most one alteration per sample. For the colon cancer dataset, 1514 gene-sets had at least one alteration, 1047 of which had at most one alteration per sample.

We compare the four patient-oriented methods we introduced to the gene-oriented method which uses the LRT gene-specific scores and the Wilcoxon test by using CAT plots (described in the main portion of the manuscript) in Figure 1, 2, and 3 below. As in the case of the glioblastoma dataset, we note that any two of the patient-oriented methods have a much higher fraction of top-ranked sets in common than the gene-oriented method and one of the patient-oriented methods. The plots display just two of the patient-oriented methods for the comparison to the gene-oriented method, for clarity, but the remaining two comparisons are very similar. We note that the differences between the patient-oriented and gene-oriented methods become somewhat more attenuated for the breast and colorectal tumors, as the number of samples is considerably lower, but they are still pronounced even in this case.

Figure 1: CAT plot comparing the patient-oriented methods to the gene-oriented method for the pancreatic cancer data from [3].

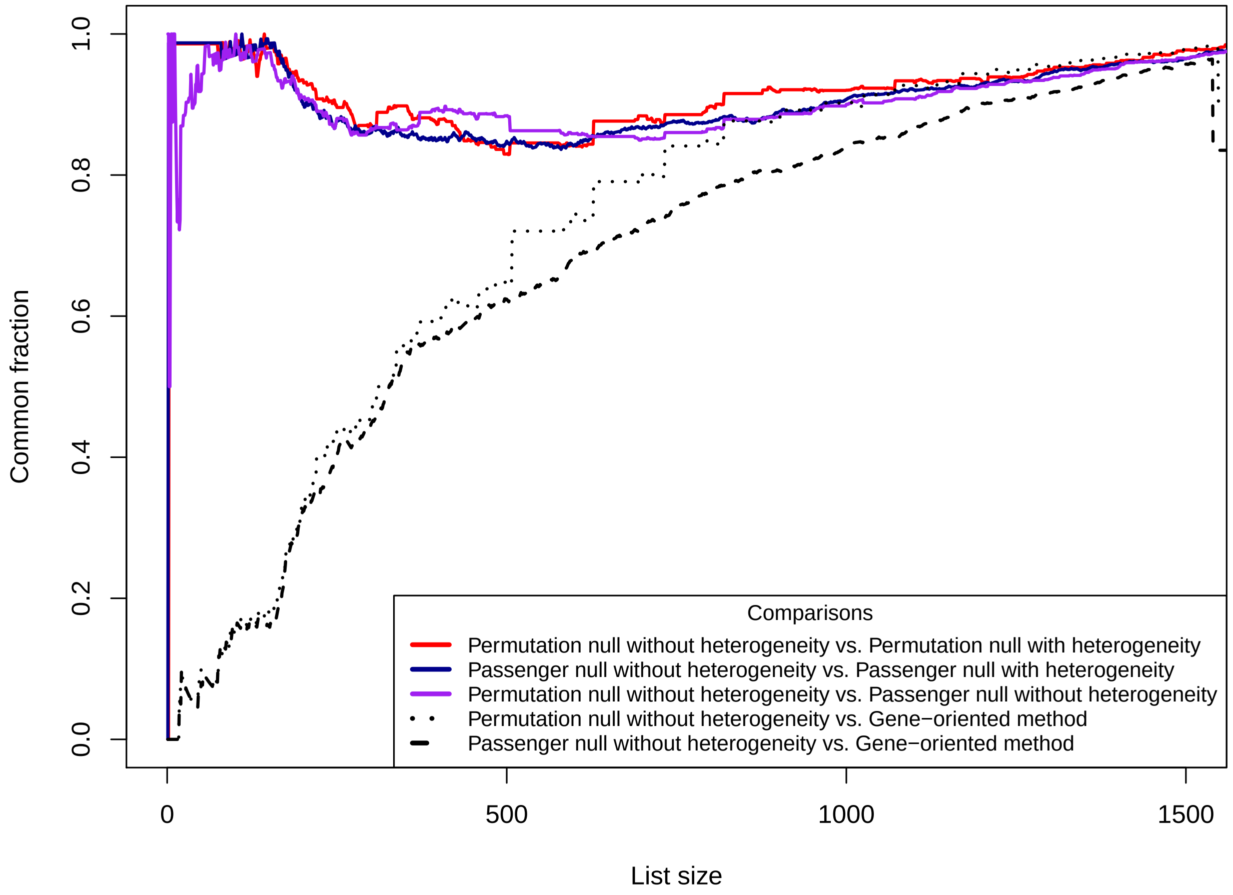

Figure 2: CAT plot comparing the patient-oriented methods to the gene-oriented method for the breast cancer data from [1] and [2].

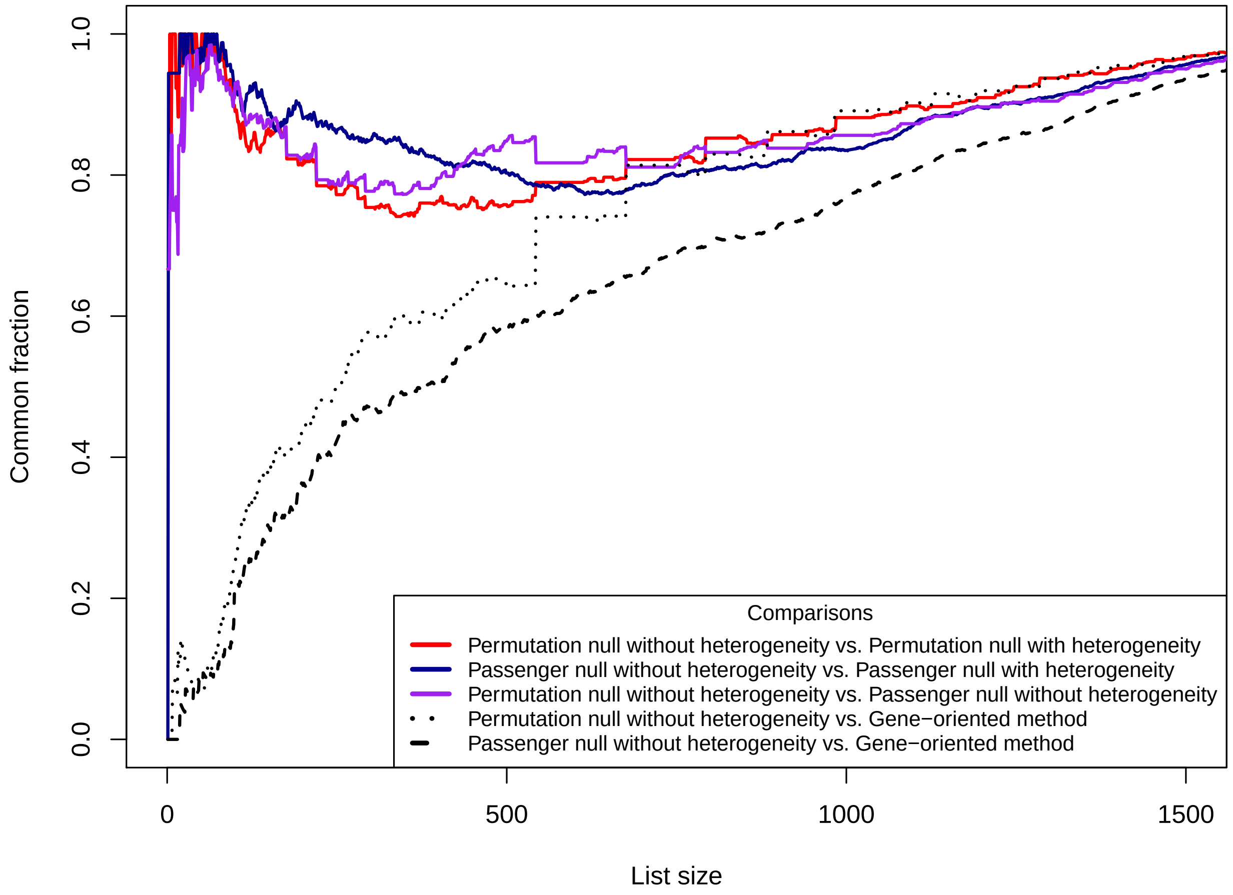

Figure 3: CAT plot comparing the patient-oriented methods to the gene-oriented method for the colorectal cancer data from [1] and [2].

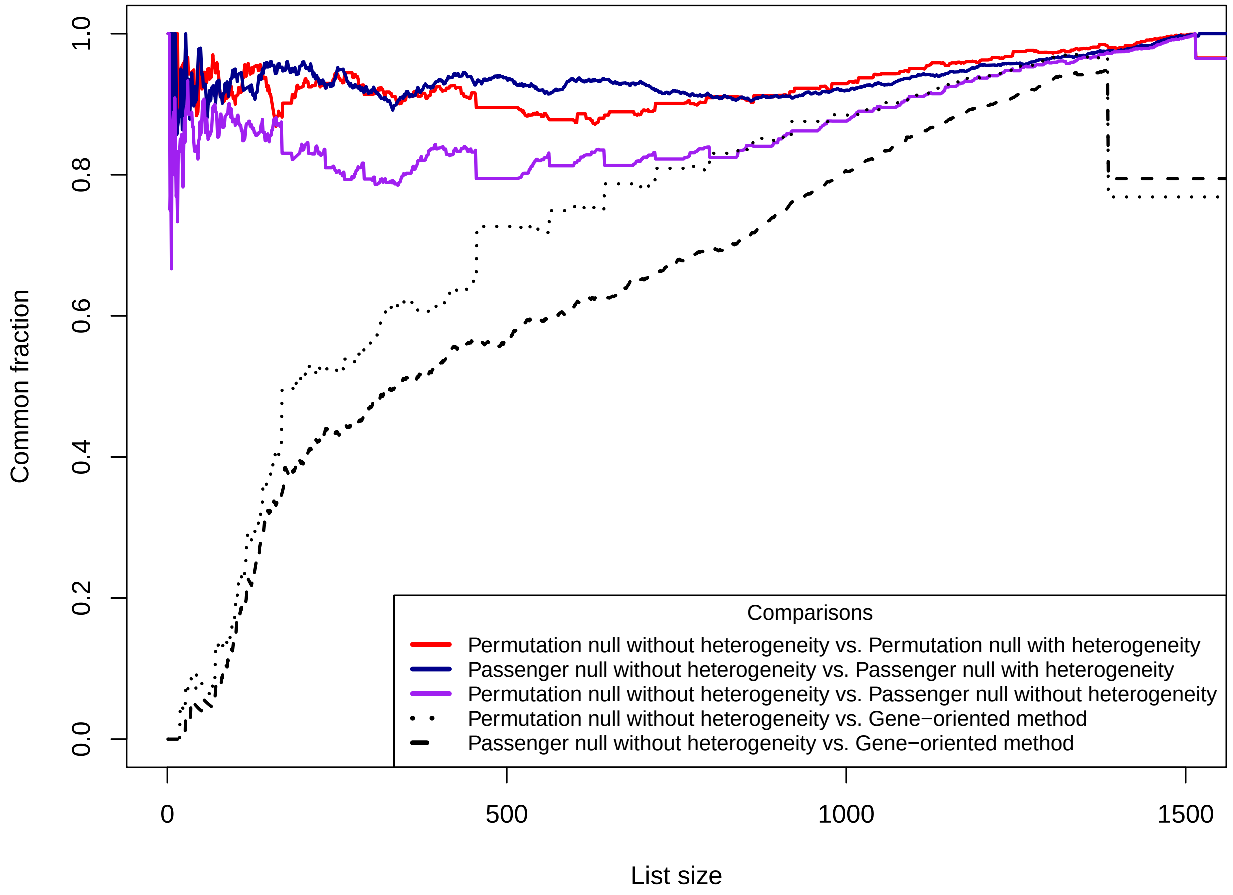

Supplement: Additional file 4 — Analyses on three more datasets. [file gb-2010-11-11-r112-S4.pdf]
